# Wireless Device Therapy in Hypertrophic Cardiomyopathy Using the Combination of a Leadless Pacemaker and a Subcutaneous Defibrillator: A Report with 2-year Follow-up of Two Patients

**DOI:** 10.19102/icrm.2024.15064

**Published:** 2024-06-15

**Authors:** Nikias Milaras, Ioannis Ntalakouras, Stefanos Archontakis, Panagiotis Dourvas, Nikolaos Ktenopoulos, Tzontatan Klogkeri, Vasileios Nevras, Skevos Sideris

**Affiliations:** 1 Cardiology Department, General Hospital of Athens Hippokrateion, Athens, Greece

**Keywords:** Cardiac implantable electronic device, leadless pacemaker, subcutaneous implantable cardioverter-defibrillator

## Abstract

Cardiac implantable electronic devices (CIEDs) are essential tools in cardiology for tackling rhythm disturbances and have come a long way over the last decades. Technology is shifting toward leadless devices that spare the complications and limitations of traditional intravascular CIEDs. Herein, we report the simultaneous implantation of a leadless pacemaker (LP) and a subcutaneous implantable cardioverter-defibrillator (S-ICD) in two patients with hypertrophic cardiomyopathy, as well as their 2-year follow-up results, while explaining the preventive measures taken to steer around unwanted device interaction. Implantation of an S-ICD with an LP is reserved for unique cases but is a feasible approach when there is a contraindication for intravascular CIED implantation. Furthermore, this technique may be used in younger patients with cardiomyopathies in whom multiple generator replacements are expected, along with their known adverse effects.

## Case presentations

### Case 1

**Figure 1:**
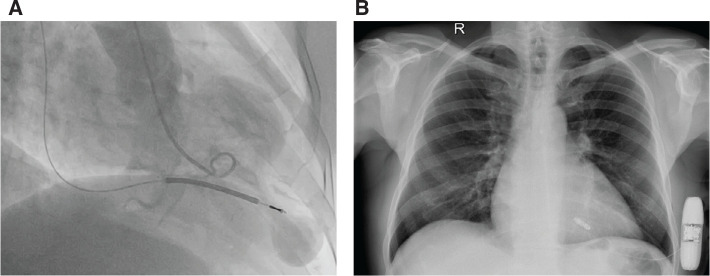
**A:** Left ventriculography in systole, revealing an apical aneurysm. **B:** A chest X-ray after implantation of a leadless pacemaker and a subcutaneous defibrillator.

A 60-year-old male patient with a medical history of an aneurysmatic sarcomeric apical hypertrophic cardiomyopathy and permanent atrial fibrillation was referred to our clinic for the evaluation of two episodes of syncope. As a first step, we performed 24-h ambulatory electrocardiographic monitoring, revealing atrial fibrillation with pauses of up to 3.5 s in duration, during wake hours, as well as short bursts of non-sustained ventricular tachycardia (VT). His hypertrophic cardiomyopathy risk–sudden cardiac death (HCM Risk-SCD) score was calculated to be 7.6%; therefore, we decided to proceed to transvenous defibrillator (implantable cardioverter-defibrillator [ICD]) implantation, considering the syncope to be of an arrhythmic etiology **(Figure 1A)**.

The implantation was uncomplicated; however, during the first-year regular follow-up re-evaluation, he presented with ankle edema and mild dyspnea on exertion. Transthoracic echocardiography revealed new-onset severe tricuspid regurgitation (TR) due to anterior leaflet malcoaptation from the right ventricular ICD lead.

After thorough discussion, we proceeded with the extraction of the ICD and subsequently to simultaneous implantation of a leadless pacemaker (LP) (Micra; Medtronic, Minneapolis, MN, USA) and a subcutaneous ICD (S-ICD) (EMBLEM; Boston Scientific, Marlborough, MA, USA), as the latter lacks pacing capability **(Figure 1B)**. Restricted superior vena cava access remains a main indication for the implantation of both an LP and an S-ICD and was favored in this patient diagnosed with a symptomatic iatrogenic valvular insufficiency leading to the extraction of the previously implanted transvenous device.

Lead removal, in these cases, is fraught with its own potential complications, and, in addition, it is unclear whether it will lead to the improvement of TR severity or right ventricular function.

### Case 2

**Figure 2:**
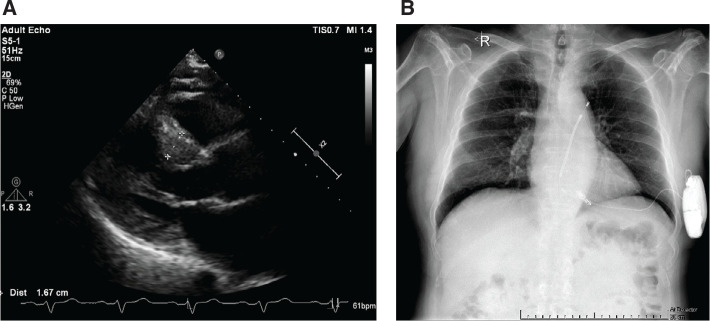
**A:** An echocardiographic parasternal short-axis view with septal hypertrophy measuring 1.67 cm. **B:** A chest X-ray after implantation of a leadless pacemaker and subcutaneous defibrillator.

A 67-year-old female patient, with a history of typical septal HCM **(Figure 2A)** and a single-lead ICD implanted 2 years ago, presented with a fever of 10 days’ duration and enlargement of the right infraclavicular area anterior to the device generator.

The patient originally underwent a dual-chamber ICD implantation on the left infraclavicular area; however, 9 years ago, the system was extracted 2 years ago due to bacterial endocarditis caused by *Staphylococcus epidermidis*, diagnosed shortly after generator replacement. After 6 weeks of hospitalization for antibiotic therapy, she received a single-chamber ICD implanted on the right infraclavicular area. The risk for CIED-related infection is almost threefold higher after generator replacement than following the initial implantation.^1^ Most studies indicate that usually antibiotic therapy and both device and lead extraction are unequivocal, leading to an improved prognosis when performed early, at experienced centers.^1^

This patient also underwent alcohol septal ablation due to excessive left ventricular outflow tract obstruction. Atrial fibrillation developed perioperatively, which kept relapsing even after prompt pharmacotherapy and frequent cardioversions. The patient remained mildly symptomatic for the last 9 years, with device interrogation revealing small bursts of non-sustained VT that did not require ICD therapy, permanent atrial fibrillation, and ventricular pacing of 70%.

The patient was further assessed in our department, and an ICD pocket infection was diagnosed. Device extraction was planned once again, after prompt antibiotic therapy. Keeping in mind the high percentage of ventricular pacing, the need for protection from sudden cardiac death, and the successive device-related infections, we decided to proceed with simultaneous implantation of an LP (Micra; Medtronic) and an S-ICD (EMBLEM; Boston Scientific) after administration of a 6-week antibiotic scheme **(Figure 2B)**.

The patient is being followed up in our outpatient department, and no CIED-related adverse events were recorded during the last 2 years of follow-up.

## Discussion

Even though implantations of LPs and S-ICDs are viable alternatives, facilitating the implanter’s everyday practice, both devices are rarely used in tandem. The safety and efficacy of each device have been proven through large multicenter trials, while their combined characteristics have been described in just a few case reports.^2^ After reviewing the literature, the main indication for the implantation of this combination of CIEDs is prior infection.^3^ In our experience, LP implantation after intravenous CIED extraction due to infection does not lead to re-infection, and no relevant reports have been published in the literature. The rationale behind our approach is to implant the least intravenous hardware possible, as prior infection remains a major risk factor for re-infection.

TR, on the contrary, was the reason for intravenous CIED extraction in case 1. It is a well-known complication, occurring mainly due to leaflet perforation, avulsion, or damage of the subvalvular apparatus. Leaflet adhesion, fibrosis, and encapsulation may further contribute to valve incompetence.^4^

TR is considered an independent risk factor for all-cause mortality and unfortunately leads to a vicious circle, enhancing right ventricular enlargement, further TR, and eventually right ventricular failure. Secondary TR usually improves with diuretic therapy, although medical therapy has little effect in the presence of primary valvular disease.

In order to prevent inappropriate shocks or unexpected device interaction, we usually take the following measures.

After implantation, we test the defibrillation capacity of the S-ICD at 80 J after inducing ventricular fibrillation (VF). Even though the LP is not inhibited during VF and pacing spikes resume during the arrhythmia, the S-ICD tracks the difference in amplitude and frequency on electrocardiography and delivers appropriate therapy. In our experience, no LP malfunction or visible translocation occurred after defibrillation.

Another key concern with S-ICD and LP combined therapy is that pacing spikes and QRS components might be oversensed by the S-ICD and may interfere with ventricular arrhythmia detection algorithms.^5^ To tackle this, we simultaneously perform LP and S-ICD interrogation in both supine and standing positions before discharge. We try to perform this while pacing through the LP and without pacing when possible. Then, the best S-ICD vector is chosen in order to avoid T-wave oversensing. Furthermore, the S-ICD is capable of storing and recognizing the templates of both the native and paced QRS complexes.

This device combination widens our therapeutic arsenal, especially in the sicker patient with prior infective endocarditis or limited upper limb venous access. Even though adverse events have yet to occur in our two patients, this strategy has not been formally validated. A combination of such devices with cross-talk capability would be an ideal alternative.

## Conclusion

This miniature case series highlights the possibility of using two independent devices—namely, the LP and S-ICD—in two different scenarios of HCM patients.
